# Market access of Chinese patent medicine products to healthcare security system in China: implications for international integration of traditional medicine into health systems

**DOI:** 10.1186/s13020-021-00560-w

**Published:** 2022-01-04

**Authors:** Chenglai Xia, Dongning Yao, Yunfeng Lai, Yan Xue, Hao Hu

**Affiliations:** 1 Southern Medical University Affiliated Maternal & Child Health Hospital of Foshan, Foshan, 528000 China; 2 School of Pharmaceutical Sciences, Southern Medical University, Guangzhou, 510150 China; 3 State Key Laboratory of Quality Research in Chinese Medicine, Institute of Chinese Medical Sciences, University of Macau, Macao, SAR China; 4 School of Public Health and Management, Guangzhou University of Chinese Medicine, Guangzhou, China; 5 Department of Public Health and Medicinal Administration, Faculty of Health Sciences, University of Macau, Macao, SAR China

**Keywords:** Chinese patent medicine, Chinese medicine, Market access, Healthcare security system, Price negotiation, Pharmacoeconomics

## Abstract

**Background:**

China has introduced a series of polices and practice to manage the market access of Chinese patent medicine (CPM) products into its healthcare security system, which is less analyzed and reported in current literature. Therefore, this paper aimed to investigate the mechanisms managing market access of CPM products into healthcare security system in China, expecting to provide implications for international integration of traditional medicine products into health systems.

**Method:**

This paper used a documentary analysis approach as a qualitative research method. Data were collected from four sources and analyzed in a thematic way.

**Results:**

Four mechanisms to manage entry, price adjustment, and exit of innovative brand and generic CPM products are identified, including: (1) price negotiation, mechanism of new entry of innovative brand CPM products into the national reimbursement list; (2) price re-negotiation, mechanism of price adjustment of innovative brand CPM products within the national reimbursement list; (3) mass procurement, mechanism of generic CPM products to healthcare security system; and (4) direct removal, mechanism of removal from the national reimbursement list.

**Conclusions:**

China has established market access framework of CPM products by focusing on price negotiation for innovative brand CPM products and mass procurement for generic CPM products. Further studies of CPM products based real-world data are needed to provide clinical and pharmacoeconomic evidence to support market access of CPM products into healthcare security systems.

## Background

With the application of modern science and technology into development of traditional medicine product, public acceptance of traditional medicine products is continuously increasing [1, 2]. However, traditional medicine products still face extraordinary challenges of market access into healthcare security systems [3–5]. In particular, compared with the relatively developed market access institutions for conventional medicine products, institutional arrangement of market access of traditional medicine products remains blank in most healthcare security systems [6].

China is one of the countries that have widely used traditional medicine products in their healthcare security systems [7]. Chinese patent medicine (CPM) products are often applied for treatment and maintenance since the foundation of the People’s Republic of China in 1949 [8, 9]. But there were no standardized regulations for managing market access of CPM products into healthcare security system for a long period. The health departments mostly rely on historical utilization tradition and experts’ opinions to decide the inclusion or exclusion of CPM products for healthcare security, which raised much criticisms from the public and industry [10].

To meet the challenges of regulating market access of CPM products, the National Healthcare Security Administration (NHSA) of China and the related ministries such as the National Medicinal Product Administration (NMPA) and the National Health Commission (NHC), etc., had introduced a series of policies and experimented some innovative practices in the past years. With these reforms in market access regulations, market access of CPM products has been mostly operated in an institutionalized routine. On 28th December 2020 the NHSA officially publicized the “National Basic Medical Insurance, Work Injury Insurance and Maternity Insurance Drug List (2020)” (so-called the national reimbursement list (2020)). Among the 2,800 drugs included into the reimbursement list, there are 1,315 CPM products, showing a significant access of CPM products to the healthcare security system. However, these policy and regulation changes for market access of CPM products into healthcare security system have not been systematically analyzed and reported in current literature.

Therefore, in this paper, we aimed to investigate the mechanisms managing market access of CPM products into healthcare security system in China. It is expected that the findings can generate evidence for future optimization of market access policies for CPM products in China. Also, it is expected that the findings can provide references for international innovation of traditional medicine and integrating traditional medicine products into health systems.

## Method

### Research design

To understand the inner content and logic of market access framework of CPM products into healthcare security systems in China, this paper used a documentary analysis approach [11] as a qualitative research method [12]. This research design is reviewed and approved by the University of Macau (MYRG2020-00230-ICMS).

### Data collection

For data collection, all the documentary materials were collected from four sources. First, government documents were collected from the NHSA (http://www.nhsa.gov.cn), the NMPA (http://www.nmpa.gov.cn) and the NHC (http://www.nhc.gov.cn) as three key ministries that are involved in designing and implementing market access polices. Except direct search on their official websites, we have reviewed the annual policy collections published by the ministries since 2018 when the NHSA was officially founded.

Second, we collected formal documents of market access publicized from the CPM manufacturers that have supplied CPM products according to the reimbursement list (2020). Third, we searched the China National Knowledge Infrastructure to identify academic publications about the market access of CPM products since 2018. Fourth, we searched online through Baidu search engine (http://www.baidu.com) to collect industrial analysis reports about market access of CPM products released by investment banks. Such kind of diversified strategies ensure that we can collect sufficient documents to meet the criteria of data saturation and to conduct triangular test for data validity.

### Data analysis

For data analysis, we followed the thematic analysis method proposed for qualitative data analysis [13]. First, after familiarizing with all the materials, we analyzed the materials by dividing two types of CPM products: innovative brand CPM products and generic CPM products. Second, considering the whole life cycle CPM products within the healthcare security system, we analyzed the market access mechanisms of CPM products from three aspects: entry, price adjustment, and exit. Finally, mechanisms (including applicable criteria and processes) for these two types of CPM products were identified respectively.

## Results

**Fig. 1:**
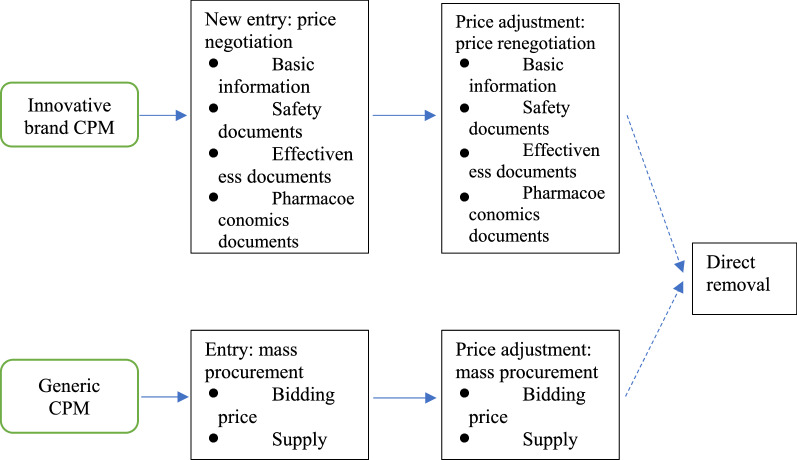
Market access of CPM products: innovative CPM vs. generic CPM

The mechanisms of the access, price adjustment and exit for innovative CPM and generic CPM can be summarized as Fig. 1. Each mechanism is presented in details as below.

### Mechanism of new entry of innovative brand CPM products into the national reimbursement list: Price negotiation

For innovative brand CPM products (or so-called exclusive CPM products in China), which usually have no competitor drugs and have not been included into the reimbursement list before, the NHSA established the new entry mechanism to manage the access application of innovative brand CPM products. The focus of this new entry mechanism is price negotiation.

To apply for price negotiation, the CPM firms need to submit a series of safety, efficacy (effectiveness) and pharmacoeconomics documents about their innovative brand CPM products, including:

- Basic information (such as general name, time to market at home country and abroad, main indications, brief usage and dosage, and indications to be negotiated for access, etc.);
- Safety documents (such as RCT or adverse reactions in the real-world, etc.);
- Effectiveness documents (such as main clinical outcome indicators, differences in efficacy (effectiveness) compared with similar drugs or therapies, and clinical guidelines recommendations, etc.);
- Pharmacoeconomics documents (such as overall results of cost-effectiveness analysis, overall results of budget impact analysis, current domestic and foreign prices, charity donation plans, comparison with reference drug prices and total direct medical cost, and domestic and foreign medical insurance access, etc.);

After receiving the application documents, the expert panels organized by the NHSA will evaluate the clinical and pharmacoeconomics documents submitted by the CPM firms. Moreover, the expert panels will carry out evidence-based pharmacy evaluation, cost-effectiveness analysis, and budget impact analysis by themselves and generate independent report and recommended price.

With the independent report and recommended price, the representatives of the NHSA will meet the representatives of the CPM firms fact-to-face to negotiate the price. If the price proposed by the CPM firms fall into the range of the recommended price set by the expert panels, a final reimbursement price will be reached. Otherwise, the price negotiation will fail and the innovative brand CPM product will not be included into the national reimbursement list.

### Mechanism of price adjustment of innovative brand CPM products within the national reimbursement list: Price re-negotiation

Except the mechanism of new entry to include innovative brand CPM products into the national reimbursement list, the NHSA realizes that it also needs to dynamically manage the innovative brand CPM products that have been listed in the national reimbursement list. Therefore, the NHSA established the mechanism of price change to implement dynamic adjustment of price or payment scope of innovative brand CPM products within the national reimbursement list. According to the regulation of the NHSA, such kind of mechanism is applied for three types of situations, including:

1. Negotiated drugs that are within the valid period of the agreement and need to re-determine the payment standard in accordance with the agreement.
2. Drugs that are necessary to adjust the scope (treatment field) of payment, according to the firm application or expert evaluation.
3. Drugs whose prices/expenses are obviously higher and that have taken up a large amount of healthcare security funds in recent years, compared with other drugs in the same treatment field.

The focus of such mechanism is price re-negotiation. The whole process is similar with that of new entry of innovative brand CPM products: first, CPM firms need to submit documents about basic information, safety, effectiveness, and pharmacoeconomics evaluation, et; second, the NHSA will organize expert panel to conduct independent evaluation to generate evaluation report and recommended price; third, the NHSA representatives and firm representatives will have face-to-face negotiation to reach an agreement price if negotiation succeeds.

For example, in 2020 six CPM products within the national reimbursement list have proceeded price change mechanism to remain in the national reimbursement list (see Table 1). Before re-negotiation, each of the six CPM products had cost more than 1 billion CNY in the healthcare security system every year. Through price re-negotiation, it is expected the national healthcare security system can save about 50% funding for the six CPM products.

**Table 1:** CPM products re-negotiated within the national reimbursement list (2020)

	Name	Drug classification	Ingredient	Dosage	Indication	Price in 2020 list
1	Kanglaite injection	Dispel syndromes and dispel masses	Coix Seed Oil	Injection	Primary non-small cell lung cancer and primary liver cancer	• 136 CNY (100 ml:10 g)
2	Kangai injection	Replenish vital energy and strengthen the body’s immune function	Astragalus, ginseng, matrine	Injection	Primary liver cancer, lung cancer, rectal cancer, malignant lymphoma, gynecological malignancy	• 11.7 CNY (5 ml) • 19.94 CNY (10 ml) • 33.9 CNY (20 ml)
3	Salvianolate injection	Invigorate blood circulation, remove blood stasis, and relieve pulse	Salvia miltiorrhiza polyphenolate	Injection	Stable angina pectoris	• 31.69 CNY(50 mg) • 53.88 CNY (100 mg) • 91.60 CNY (200 mg)
4	Danhong injection	Promote blood circulation and remove blood stasis	Salvia, safflower	Injection	Chest pain, chest tightness, coronary heart disease	• 5.05 CNY (2 ml) • 17.32 CNY (10 ml) • 29.44 CNY (20 ml)
5	Lanqin oral liquid	Clear heat and detoxify, relieve throat and swelling	Radix isatidis, scutellaria baicalensis georgi, gardenia, cork, sterculia	Oral liquid	Acute pharyngitis, sore throat, dry throat, burning throat caused by lung and stomach heat syndrome	• 3.46 CNY (10 ml) • 5.88 CNY (10 ml)
6	Bailing Capsule	Nourish lungs and kidneys, nourish essence and qi	Fermented cordyceps fungus powder (Cs-C-Q80)	Capsule	Treatment of kidney disease, treatment of type 2 diabetes with microalbuminuria, treatment of recurrent urinary tract infections, treatment of liver diseases, treatment of respiratory diseases, adjuvant treatment of tumors, etc.	• 0.51 CNY (0.2 g) • 1.03 CNY (0.5 g)

### Mechanism of generic CPM products into healthcare security system: mass procurement for entry/price adjustment

For generic CPM products that are manufactured by several companies, mass procurement is the mechanism for them to get access to healthcare security system, including new entry into the healthcare security system and making price adjustment. In practice, responding to the call for mass procurement application publicized by the healthcare security department, manufacturers can submit their bidding documents by indicating their bidding price and supply capacity. The healthcare security department will compare the bidding price submitted and usually select the 2-3 manufactures with the lowest price as final CPM product suppers.

Different from the centralized drug procurement organized by the central NHSA for generic chemical drugs, currently mass procurement for generic CPM products is only organized at provisional or city level. The reason is that the NMPA has only founded the consistency evaluation regulation for generic chemical drugs. Because CPM products still face the challenges of clarifying composition, the NMPA is still unable to organize consistency evaluation for CPM products. Consequently, the NHSA decided to leave the mass procurement to provisional level or city healthcare security departments.

### Mechanism of exit from the national reimbursement list: direct removal

For the CPM products that have been included into the national reimbursement list, the NHSA also established the mechanisms of direct removal to enable dynamic adjustment to eliminate the unsuitable drugs. Such kind of mechanism applies for two kinds of conditions:

1. Drugs of which the drug approval documents have been revoked or cancelled by the NMPA;
2. Drugs of which the risk is considered to be greater than the benefit after comprehensive evaluation of their clinical value, adverse reactions, pharmacoeconomic value, and other factors.

To implement of the exit mechanism, the NHSA annually scans the reimbursement list with reference to the official announcement of the NMPA, the reimbursement data of provisional SSAs, the adverse reaction reports from the NMPA and hospitals, etc., in order to identify the CPM products of potential concerns. Then, the NHSA will organize specific expert panel which may compose of clinical experts, clinical pharmacy experts, and pharmacoeconomics experts to carry out systematic evaluation based on the current evidence available without any input from the CPM firms. If the final benefit-risk report is negative, the CPM product will be directly removed from the reimbursement list without interactions with the CPM firms.

For example, in the national reimbursement list (2000), three CPM products were directly removed through such mechanism (see Table 2). *Coptis Phellodendron Burn Ointment* and *Ginseng Astragalus Eleven Flavor Tablets/Capsules* were removed because the manufacturers no longer existed. While *Loquat Leaf Cream* still has 15 production approvals, it is directly removed because its clinical value is deemed inadequte and it can be replaced by other drugs with equivalent or better curative effect in the reimbursement list.

**Table 2:** CPM products directly removed from the national reimbursement list (2020)

	Name	Drug classification	Ingredient	Dosage	Indications	Production approval
1	*Loquat Leaf Cream*	Nourishing lungs and reducing phlegm	Loquat leaves, supplemented with sucrose	Ointment	Lung heat cough, Less phlegm and dry throat	15
2	*Coptis Phellodendron Burn Ointment*	Antipyretic and antidote	Coptis, Phellodendron, Garcinia (made), Borneol	Ointment	Used for the treatment of superficial and deep second-degree burn wounds, the area of medication should not exceed 3% of the body surface area	0
3	*Ginseng Astragalus Eleven Flavor Tablets/Capsules*	Invigorate the spleen	Ginseng, Astragalus, Angelica, Gastrodia, Rehmannia, Alisma, Cassia, Antler, Cuscuta, Asarum, Chinese wolfberry, etc.	Tablets/capsules	Used for the leukopenia caused by radiotherapy and chemotherapy in cancer, and dizziness, dizziness, fatigue, weight loss, nausea, vomiting and other symptoms caused by radiotherapy and chemotherapy.	0

## Discussion

Through systematic documentary research, this paper identified four mechanisms that are applied to regulate the market access of CPM products to healthcare security system in China, which are not critically reported in the past literature. These findings raise some issues that are worth further discussion for innovative brand CPM products and generic CPM products respectively.

For innovative CPM products, it shows that the NHSA of China is trying to found a value-based market access framework for CPM products. For innovative conventional medicine products, developed countries have established a market access framework that depends on health technology assessment and price negotiation [14, 15]. But there is no such a mature framework for traditional medicine products until now. In China, referring to the market access framework for innovative conventional medicine products, the NHSA is trying to apply an evidence-based price negotiation for innovative brand CPM products. Thus, the NHSA requires manufacturers to submit their evidence documents, conducts internally independent assessment, and then organize face-to-face price negotiation to decide the final market access for innovative brand CPM products. The market access framework for innovative brand CPM products by the NHSA then provides a starting mode for future improvement of market access for traditional medicine product internationally.

However, it is noteworthy that there are two key differences between innovative conventional medicine products (chemical drugs and biopharmaceutical drugs) and innovative brand CPM products though the general administrative processes of market access are the same. Firstly, while the major source of evidence about the efficacy of innovative conventional medicine products relies on randomized controlled trials, the effectiveness of the innovative brand CPM products is primarily based on real-world data. Due to the limitations in conducting randomized controlled trials with innovative brand CPM products, the effectiveness evidence of innovative brand CPM products based on real-world data is permitted and accepted [16, 17]. Secondly, regarding pharmacoeconomic evidence, while innovative conventional medicine products usually need to choose the competing products in the same category as comparators, innovative brand CPM products generally are required to choose the competing conventional medicine products for the same disease indication as comparators for pharmacoeconomic evaluation purposes. In general, it shows that amid the methodological challenges for innovative brand CPM products, the authorities of healthcare security systems are trying to develop alternative ways for assessing market access of innovative brand Chinese medicine.

For generic CPM products, it shows that the NHSA of China is experimenting to establish an institutional market access framework for generic CPM products through price competition on the basis of quality assurance. This market access framework is different from that of innovative brand CPM products, because generic CPM products have usually been approved and used clinically for years, which has demonstrated their effectiveness and safety. As general CPM products usually have several manufacturers to supply, bidding price often play the decisive role in final market access results. However, quality assurance will be the prerequisite factor for market access of generic CPM products, particularly considering the effects of manufacturing processes on CPM product quality [18]. In China, while quality standards for generic CPM products have been included into the China Pharmacopeia, the NMPA of China has not organized the consistency evaluation for generic CPM products yet [19]. Because of the scientific and technological challenges in evaluating CPM products, how to conduct consistency evaluation for generic CPM products remains debating [20–22]. Future development of consistency evaluation for generic CPM products will affect the design and operation of market access framework for generic CPM products in the long run.

While this paper mainly focuses on market access of CPM products into healthcare security system in China, the findings of this paper also have several implications for international integration of traditional medicine products into health systems. First, there should be separated market access frameworks for innovative traditional medicine products and generic traditional medicine products. Considering the different innovation nature and evidence accumulation, such kind of separated arrangement for innovative and generic traditional medicine products is necessary. Second, systematic evaluation including efficacy (effectiveness), safety and economic elements should be pre-conditions for market access of traditional medicine products. Notably, real-world evidence needs to be encouraged and applied for traditional medicine products [23]. Third, pharmacoeconomic evaluation of traditional medicine products should be an indispensable part of market access of traditional medicine products [24, 25]. Considering the pharmacoeconomic value of traditional medicine products from the perspective of payers will pave the way of traditional medicine products for smoother market access internationally [26].

To our knowledge, this is the first paper that studies the market access of CPM products into healthcare security system. However, there are some research limitations that can be addressed in future studies. First, this papers only used the documentary materials for analysis. As practitioners such as the NHSA officials, industrial management and clinical experts, etc., have their own idea and judgement in the realistic practice of CPM market access, future studies could conduct qualitative interviews with first-line practitioners to collect their opinions about the barriers and suggestions for improving market access of CPM products. Second, mass procurement for generic CPM products is only experimentally operating at provincial or city level. Future studies on mass procurement of generic CPM products to establish a formal framework of centralized mass procurement is necessary. Third, the market access to healthcare security system is different from the access to hospital. Particularly in China, entry into the national reimbursement list does not guarantee direct access to individual hospital formulary. How to promote the inclusion of CPM products on the national reimbursement list into the individual hospital formulary is another important but complex topic. Future study focuses on enlistment of CPM products into hospital formulary is needed. Fourth, it is necessary to evaluate the long-term impact of the CPM market access policies. In particular, mathematical modelling methods based on first-hand real-world data should be developed to help assess and optimize the market access policies for CPM products.

## Conclusions

China has established market access framework of CPM products by focusing on price negotiation for innovative brand CPM products and mass procurement for generic CPM products. Further studies of CPM products based real-world data are needed to provide solid clinical and pharmacoeconomic evidence to support market access of CPM products into healthcare security systems.

## Data Availability

All data generated or analysed during this study are included in this published article.

## Abbreviations

CPM: Chinese patent medicine
NHC: National Health Commission
NHSA: National Healthcare Security Administration
NMPA: National Medicinal Product Administration
